# 

**Published:** 1942

**Authors:** 


### CONTENTS.

*Page*

Inunction of Testosterone in Mental Disease. By A. Guirdham, D.M., B.Sc., D.P.M. .. .. .. .. .. .. .. 1

The Fundamental Principles of Fracture Treatment. By K. H. Pridie, F.R.C.S... .. .. .. .. .. .. .. 7

Experiences of Typhus Fever. By F. Kohn, M.D. .. .. 13

The Bristol Scheme for Dealing with an Outbreak of Typhus. By R. J. Irving Bell, M.R.C.S., L.R.C.P., D.P.H. .. .. .. 16

Anæsthesia .. .. .. .. .. .. .. .. .. 19

Reviews of Books .. .. .. .. .. .. .. .. 23

Editorial Notes .. .. .. .. .. .. .. .. 27

The Medical Library of the University of Bristol.. .. .. 33

Publications Received .. .. .. .. .. .. .. 38

Local Medical Notes .. .. .. .. .. .. .. 39

